# The hospital-to-home care transition experience of home care clients: an exploratory study using patient journey mapping

**DOI:** 10.1186/s12913-023-09899-2

**Published:** 2023-08-31

**Authors:** Marianne Saragosa, Sonia Nizzer, Sandra McKay, Kerry Kuluski

**Affiliations:** 1 Lunenfeld-Tanenbaum Research Institute, Sinai Health System, 1 Bridgepoint Drive, Toronto, ON M4M 2B5 Canada; 2 VHA Home HealthCare, Toronto, ON Canada; 3 Department of Physical Therapy, University of Toronto, Toronto, ON Canada; 4 Ted Rogers School of Management, Toronto Metropolitan University, Toronto, ON Canada; 5 The Institute for Education Research, University Health Network, Toronto, ON Canada; 6 Michael Garron Hospital, Toronto East Health Network, East York, Toronto, ON Canada; 7 Trillium Health Partners, Mississauga, ON Canada; 8 Institute of Health Policy, Management and Evaluation, University of Toronto, Toronto, ON Canada

**Keywords:** Care transition, Home care, Journey mapping, Patient experience, Caregiver experience

## Abstract

**Background:**

Care transitions have a significant impact on patient health outcomes and care experience. However, there is limited research on how clients receiving care in the home care sector experience the hospital-to-home transition. An essential strategy for improving client care and experience is through client engagement efforts. The study's aim was to provide insight into the care transition experiences and perspectives of home care clients and caregivers of those receiving home care who experienced a hospital admission and returned to home care services by thematically and illustratively mapping their collective journey.

**Methods:**

This study applied a qualitative descriptive exploratory design using a patient journey mapping approach. Home care clients and their caregivers with a recent experience of a hospital discharge back to the community were recruited. A conventional inductive approach to analysis enabled the identification of categories and a collective patient journey map. Follow-up interviews supported the validation of the map.

**Results:**

Seven participants (five clients and two caregivers) participated in 11 interviews. Participants contributed to the production of a collective journey map and the following four categories and themes: (1) Touchpoints as interactions with the health system; *Life is changing*; (2) Pain points as barriers in the health system: *Sensing nobody is listening* and *Trying to find a good fit*; (3) Facilitators to positive care transitions: *Developing relationships and gaining some continuity* and *Trying to advocate, and* (4) Emotional impact: *Having only so much emotional capacity*.

**Conclusions:**

The patient journey map enabled a collective illustration of the care transition depicted in touchpoints, pain points, enablers, and feelings experienced by home care recipients and their caregivers. Patient journey mapping offers an opportunity to acknowledge home care clients and their caregivers as critical to quality care delivery across the continuum.

## Background

In 2021 nearly 921,700 Canadian households reported accessing formal home care [1]. The general population uses home care services for various needs, such as recovery after hospital discharge, supporting end-of-life care, or managing chronic conditions, disabilities, or mental illnesses. Internationally, variations exist within and between countries in home care organization, policies, and availability of services [2], and targeted population groups within home care systems [3, 4]. With the population aging and citizens living with disability, reliance on the home care system in combination with substantial care by unpaid caregivers enables people to live safely in their own homes. While most home care recipients (hereafter referred to as ‘client’) are 65 years and older, home care services are also provided to adults with long-term disabilities younger than 65 years [1].

Hospitalization and the period immediately following hospital discharge are particularly critical periods. The Canadian Institute for Health Information reports that 9.3% of patients discharged from the hospital are readmitted within 30 days [5]. Factors cited as predictors of readmission include hospital length of stay, patient acuity, and comorbidity [6]. Patient-reported challenges that arise during the hospital-to-home care transition require further exploration.

Understanding patient experience is important and is increasingly recognized as a quality measure, clinical effectiveness, and patient safety [7]. Patient experience is a multifaceted concept that spans a range of patient health setting/environment experiences, including lived and care experiences, clinical interactions, organizational features of care, and process measures [8]. Despite more attention on patient experience, it remains understudied in the formal home care sector [9]. The care transition experience in home care clients also requires more attention [10]. Client engagement efforts are an important strategy for improving client care and experience. Client engagement describes a partnership of clients, families, and health care professionals working together to improve the client experience [11]. Previous home care research has reported on client interest in direct care and care planning and less so on broader organizational improvement efforts [11]. However, through structured processes and engaging with older adults as experts in their lived experiences it is possible for community-dwelling service recipients to be partners in bettering health care services [12].

Journey mapping is a research approach that evolved from the market research industry to gain insight into how patients navigate and experience complex health services and systems [13–15]. As a “patient-oriented” activity, patient journey mapping is undertaken to understand the barriers, facilitators, experiences, and interactions with services or providers for those entering, navigating, and exiting a health system by documenting and producing an illustrated map [13]. Of the existing literature, patient journey mapping has been effectively used to understand the patient experience [16–18], improve the quality of care [7, 19], and for informing health service redesign/improvement [13, 15, 20]. Journey maps go beyond a static view of patients' perspectives by illustrating key moments in a patient’s journey, including important touchpoints, pain points, and the emotions experienced [7, 21]. For home care clients, mapping their journey from the hospital to home in a concise and visually compelling story may shed light on the multi-level barriers and challenges that they face, and has the potential to inspire new initiatives that improve the client experience. Using patient journey mapping, this study aimed to understand and characterize the care transition experiences for home care clients and unpaid caregivers after they transition from hospital to home with home care services. The objective of this study is to use patient journey mapping to characterize home care clients and caregivers’ experiences of home care services after transitioning from hospital to home.

## Methods

### Study design and setting

To achieve this aim, we used a qualitative descriptive exploratory design with a patient journey mapping approach. The study involved a large-scale not-for-profit charitable organization providing home care and support services in select Southern Ontario urban regions in Canada. For the purposes of this study, we recruited clients being served by the home care agency within one of the urban regions that provides both in-home personal and nursing care services. The Health Sciences Research Ethics Board of the University of Toronto (REB Human Protocol #31,494) provided ethics approval for this study.

### Participants and data collection

We applied a purposeful sampling strategy using pre-determined selection criteria to identify and recruit potential participants. We approached clients if they met the following inclusion criteria: (1) recipient of home care services from the aforementioned organization during the study period; (2) experienced a hospital admission and transitioned back home with home care upon discharge in 2021; (3) living in the community (i.e., private home, retirement home or assisted living setting that offers support services to help maintain independence); (4) aged 18 or older; (5) ability to provide informed consent and communicate verbally; (6) were not receiving palliative care; and (7) the ability to communicate in conversational English. Similarly, caregivers could be involved if they could communicate in English and self-identify as unpaid or privately paid caregivers to a home care client who met the abovementioned inclusion criteria. Unpaid family and friend caregivers and privately paid caregiver supports were required to be working with older adults living independently in their home [22]. Caregivers were recruited after clients were enrolled in the study using the same targeted measures as clients. We targeted only clients who resumed home care services, given that this enabled us to identify and invite them to participate in the study.

Three recruitment strategies were used including: social media, digital, and mailout. First, the home care organization’s client digital newsletter promoted the study through an electronic post encouraging interested clients to contact the researcher. Second, a master list of clients that were previously admitted to hospital and resumed home care services between January 1, 2021, to November 15, 2021, was generated from the organization’s administrative database. A study invitation letter was sent to potential study participants either electronically or by mail using the master list of clients, depending on the availability of the email address on file, starting with the most recently documented hospital holds in November 2021. In keeping with previous literature on hospital-to-home transitions, the aim was to conduct initial interviews between two and four weeks post-discharge [23]. A longer timespan between hospital discharge and the first client interview (i.e., > 45 days) could negatively impact clients’ ability to recall events [24]. Prospective participants contacted the researcher via email or phone to share their interest in the study. Once it was determined that potential participants met the inclusion criteria, they received the consent form for their review by email and provided informed verbal consent prior to data collection.

The study team delineated two distinct data collection phases:

- *Phase 1: Initial interview*: During the initial interview, the first author (MS) verbally collected demographic information and asked questions about the home—hospital—home care trajectory. Using a semi-structured interview guide, interview questions covered topics such as the precipitating factors to the care transition, what worked well and what was missing in terms of services and how care was delivered, and touchpoints with providers and services along the way. Examples of patient journey mapping interview guides were used to develop interview questions [13, 25, 26]. Further, the interview guide was piloted with a client partner with lived experience transitioning from hospital-to-home to ensure the questions were clear and relevant. As themes began to emerge during interviews, the semi-structured interview questions were modified using probing questions to gather more in-depth descriptions of emerging themes.
- *Phase 2: Follow-up interview*: Interviews were analyzed after the first interview and findings from phase 1 were used to create a patient journey map. The same participants were then invited to participate in a follow-up interview to review the journey map [aggregated client-level data], validate its content, suggest changes, and discuss how challenges along their journey could be addressed. If they agreed to participate in the second interview, they received the draft patient journey map through email or mail beforehand.

## Data analysis

Three of the initial participants were lost to follow-up resulting in eleven interviews, conducted from April 2022 to August 2022, which were audio recorded and transcribed verbatim. Interviews ranged from 46 to 108 min, with an average length of 52 min. Interview transcripts were analyzed iteratively using content and thematic analysis [27] to generate a collective patient journey map. We used an inductive 3-phase approach including: preparation (reading data thoroughly, immersion), organizing (open coding, developing a coding scheme, grouping the data), and reporting (generating themes reflected in the patient journey map and a conceptual understanding of the phenomenon) [27]. Members of the study team (MS, SN) with qualitative research and clinical experience worked together to develop a coding scheme by identifying broad open codes, such as “touch point,” “pain point,” “facilitator,” and “feelings.” The agreed upon coding scheme was used to analyze transcripts and develop a codebook with the broad codes and associated narratives. From this document, we developed the collective patient journey map. Further data grouping occurred, enabling us to identify overarching categories and themes from the clients’ and caregivers’ perspectives.

### Rigor

Strategies to increase the quality of our research process are collectively known as an assessment of trustworthiness and are encapsulated by the following three domains [28, 29]. (1) *Credibility* techniques that were applied involved prolonged engagement with participants across two interviews, assessment of the researcher's own influence on the research process known as reflexivity, and member checking by sharing interpretations during follow-up interviews. (2) *Dependability* was enhanced by following an analytical plan and having multiple coders. (3) *Credibility* is grounded in a team of expert researchers in qualitative methods, home care delivery, and patient engagement, who frequently met to discuss the data and emerging insights.

## Results

A total of seven participants (five home care clients and two caregivers of individuals receiving home care) completed seven initial interviews, and four completed a follow-up interview for a total of 11 interviews (Table 1). Most participants identified as female (n = 5) and were on average 66 years of age (range 44 to 88 years). Caregivers (n = 2) identified as a spouse and a paid private caregiver, with neither providing respite care.

**Table 1:** Participant Demographics

Participant	Gender	Ethnicity	Age	Type of home care service	Num. years receiving home care	Level of education	Employment status
	*Client*						
**P001**	F	White/Caucasian	40–65	Personal support (PS)	6	Post-secondary	Retired
**P002**	F	White/Caucasian	40–65	PS & Nursing	1	Graduate school	Unable to work
**P003**	F	White/Caucasian	66–88	PS	8	Post-secondary	Retired
**P005**	F	White/Caucasian	40–65	Nursing	1	Graduate school	Employed
**P006**	M	White/Caucasian	66–88	Nursing	3	High school	Retired
	*Caregiver*						
**P004**	F	White/Caucasian	66–88	PS	4	Post-secondary	Retired
**P007**	M	White/Caucasian	40–65	PS	3	High school	Employed

**Fig. 1:**
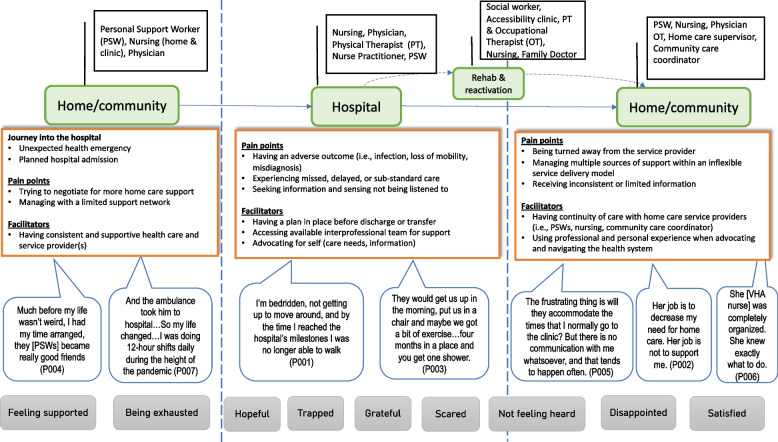
Illustration depicting the collective journey map

Participants were engaged in the production of a collective journey map (Fig. 1). The following four categories and themes emerged as key experiences in participants’ journey transitioning from hospital-to-home: (1) Touchpoints; *Life is changing*; (2) Pain points: *Sensing nobody is listening* and *trying to find a good fit*; (3) Facilitators: *Developing relationships gaining some continuity,* and *trying to advocate, and* (4) Emotional impact: *Having only so much emotional capacity*.

### Touchpoints as interactions with the health system – Life is changing

Participants described service touchpoints experienced by home care clients as they transitioned from hospital to home. Participant narratives highlight the complexity of the care transitions, including the variety of providers and multiple sectors involved. Participant 6 described:

“*A podiatrist told me the condition of my right foot did not look good and that I should probably go in to emerge and have them do an assessment. I did do that, and I wound up being admitted to the hospital, and within a couple of days, they did a partial amputation on my right foot. Then I was admitted to rehab for a two-week stay*.” (P006)

Experiences of health system touchpoints were also described by participant 004 as:

“*After 2 months of Rehab I realized if I had to do long-term care, I could but I didn’t want to, it was that simple if I could get support at home then I would, so the discharge planner started to work on how to get him support and how many times a day we would need it and that’s how we ended up with multiple agencies because we need 4 times a day*.” (P004)

While most participants described the hospitalizations as unplanned, acute situations, two individuals had planned surgeries. Regardless of the context for admission, many participants often noted that exacerbations of chronic conditions or an acute episode of illness had a life-altering impact on them or the person they cared for. For this reason, participants left the hospital setting with greater intensity in care needs than before admission. For example, one participant described the significant physical decline in coping with a hospital-acquired infection, while another explicitly noted how life had changed for her and her husband after hospital admission in an increase in touchpoints, mainly because of the pandemic,

“*We have three agencies so our life is really coo-coo compared to then when it was all just one agency and one coordinator and if you needed something special you knew exactly who to call like if you had a dental appointment you knew who to call for extra support on this day, can move this hour to here it was fine, things were more flexible.”* (P004)

### Pain points as barriers in the health system

Pain points were described as critical moments that hindered quality care transitions from the participants' perspective. Two themes emerged as *Sensing nobody is listening* and *Trying to find a good fit* and were discussed as communication and care coordination challenges.

#### Sensing nobody is listening

Several participants observed that their opinions about their treatment plan went unnoticed by the health care providers. In some cases, not being heard contributed to patient harm, including adverse outcomes and missed or delayed care. The harmful events included losing mobility and continence due to bed restrictions post-surgery, hospital-acquired infection, and a procedure-related incident. Participant 001 described feeling unheard as:

“*It was supposed to be an overnight stay, and then go home, stay overnight, one or two nights, I’ll be fine and ready to go home. It didn't turn out that way because the hospital insisted on reaching a few milestones that my body wasn't ready for, like being able to go to the toilet on my own, instead of relying on a catheter, and that takes a few days, so I’m bedridden, not getting up to move around and not getting any exercise, and by the time I reached the hospital's milestones I was no longer able to walk*.” (P001)

*“After a week he was in the hospital, maybe 10 days after, the doctor said, ‘I want to take out the stitches now.’ And I said, ‘I know [name], and I feel that maybe we could just wait a little bit.’ ‘So no. I'm taking them out. And I'm the doctor. You're not.’ So he took the stitches out and left the room, and then there were loops of small bowel coming out of the hole…So they had to rush him up to the emergency and do another operation. So that delayed everything.”* (P007)

Many participants described instances of emotional harm in the case of not receiving clear communication from health care providers and feeling frustrated and unsupported. One participant reported this experience as receiving “different stories” (P002) depending on whom you spoke to, while another individual said fragmented communication between a hospital clinic and a community provider about her leg dressing left her “removed from the situation” noted in the following quote:

“*It's probably almost like being back in high school. That's sometimes how I feel, to be honest, a couple of times I've left there in tears because it's just like nobody is listening to me. Nobody is listening to what I need. And I'm going home, and it's [leg wound] leaking all over my floor and it's frustrating not to have that communication piece. It's just like I'm there, and it doesn't matter what my experience is*.” (P005)

#### Trying to find a good fit

Most participants noted the absence of continuity of care among home care providers as a pain point when they transitioned home from the hospital. This transpired for home care clients who required more home care hours upon discharge, which could no longer be accommodated by one service provider alone. Service coverage by multiple home care agencies had participants trying to find “a good fit” (P002) as they had to coordinate not only fluctuating schedules but different home care workers and communication practices:

“*There's a definite ‘Oh, my God, here we go again, training new people.’ And we've had it throughout the year, something's happened to somebody and they've gone. And then we start with somebody new. We have not been able to get consistent care in the evening since he left. This morning, I just got a call that one of the other—that’s now the fifth agency, believe it or not, is saying they could try and pick it up in the evening and for me to call them and see what we can work out*.” (P004)

The changing service providers impacted the clients and caregivers, who often noted that they had to “train” the incoming staff about their or the person they care for personal needs. A new home care provider for some is like “starting from scratch” (P002), which has become more problematic because of COVID-19 and workforce shortages in health care:

“*The agency that was doing my PSW [personal support worker] care could not, at that time, provide me with the service, so I had to start with a new home care agency so the PSWs switched. Again, starting with new people and a new agency and retraining and trying to find a good fit. The biggest barriers have been with the agency and starting from scratch*.” (P002)

For one participant, to maintain a consistent PSW and comfort with care, the client chose to remain in her bed for 20 h to accommodate this worker’s schedule and explained her rationale for this decision in the following quote:

“*I stay in bed from 5:00 at night to 1:00 in the afternoon every Thursday, so that kind of*

*sucks, but nobody can do anything about it. I have a PSW coming at different hours every*

*day and if I want the same PSW she is not available until 1 in the afternoon. It's better for*

*me to have the same PSW because it gets exhausting to remind them to do everything*.”

(P001)

### Facilitators to positive care transitions

Facilitators are positive system and client-level factors that helped to support the transition and the adjustment to being home. This theme comprises two sub-themes that consider the importance of relationships and advocacy for quality care transitions.

#### Developing relationships and gaining some continuity

Many participants described existing relationships with home care providers as tight-knit bonds that strengthened over time. As such, these relationships were central to their care transition journey because the home care providers knew them well, including their baseline functioning, family, and household, and were often a driving force behind changes to the service plan. These longer-term relationships also enabled providers to flag medical inconsistencies among clients. For example, continuity of care in home care nursing resulted in an escalation in care because of a client’s deteriorating symptoms according to one participant,

“*She sent me back to the hospital when I was in A-fib* (heart arrhythmia) *because she knew what my normal was and she knew that this was not normal*.” (P002)

The continuity of care in service providers has also acted as a safety net for other participants who rely on one or more key people to help coordinate and deliver care in the community. One participant described an extensive network of providers: the community care coordinator, a home care supervisor, an interdisciplinary primary care program, and a personal support worker. Collectively, this team has enabled her and her husband to stay at home safely as they age.

“*I have to say that the home care supervisor has been so caring, and she's done everything that you can to get the time that we've been allotted by the care coordinator, and all it did was just reconfirmed to us that we were not going to go into an institution and that we are staying home*.” (P003)

#### Trying to advocate

All the participants reported being an advocate for themselves or the person they care for as a facilitator for a better transition experience. Advocating appeared to express their needs and requests firmly, and when supporting the participants drew from their professional skills or previous care transitions. For example, one participant managed to secure a transfer to an inpatient rehabilitation facility for additional physical therapy. Once she realized that her prior home care agency could not support her service plan, she called the home care supervisor at that agency directly.

“*I think I kind of felt that the squeaky wheel gets the worm, so to be turned away from the home care agency after having been with them for 5 years, it just floored me and scared me. To be told ‘no’ I think that was the social worker that told me, so I had to say to her ‘Hello, can you re-visit that and see if I can get back in? Rather than relying on the social worker, I phoned directly to the home care agency and spoke to my coordinator who used to schedule me.*” (P001)

Advocating for respite care through home care by one caregiver participant did not result in a service change and instead created frustration and tension for this individual. According to this participant, despite phone calls, letter writing, and an in-person meeting with the community care coordinator, their needs went unheard, and an escalation in the advocacy process seemed to be justified in this case noted in the following example,

“*So I feel I walk with integrity, and I feel she [community care coordinator] needs a wake-up call. And I will facilitate that. And it may be 20 pages, but I don't care. I've got nothing to lose. I don't care right now in my life. I will just push and push and push and push, and I'll say, ‘Well, then, I'll go to your ombudsman then. Because if you're not going to cooperate with the care plan and support me with [name’s] care, I want to make sure that everyone in the industry knows it.*" (P007)

### Emotional impact

The final category of feelings is framed by the theme, *Having only so much emotional capacity* to illustrate the emotional impact of these care transitions on clients and caregivers.

#### Having only so much emotional capacity

The participants described a mixture of primarily negative feelings when faced with unexpected challenges related to hospitalization and transition back to the community. When hospitalized, several participants felt “trapped” (P001) and “scared,” and “helpless” (P003) because of their lack of control over their situation. Once discharged from the hospital, certain feelings persisted. For example, one person expressed feeling worried about the availability of care to meet a future need based on prior experiences when,

“*My coordinator is coming out next week and because every 90 days I’m supposed to be assessed and that’s the opportunity to get support, but really her job is to decrease my need for home care…I worry because I'm due for another hospitalization to have that surgery. And I know that I'm going to come home needing more service, and I'm worried I'm not going to get it*.” (P002)

In another example, the participant called out feeling “diminished” and “worthless” when their request for an extra hour of personal support for respite went unheeded by the community care coordinator. In turn, this individual felt “taken for granted” rather than validated after many years of providing private caregiving service to a mother-son dyad,

“*So when you've been told three times in a row, ‘You're not going to get that extra hour,’ for me, it diminishes all my 27 years of work. ‘Well, I'm worthless.’ I have to go through those emotions*.” (P007)

When participants expressed positive emotions of feeling “respected” and “appreciated,” they described the enabling factors as sensing that the providers were invested in their care and wellbeing, having their questions answered and listened to, and being engaged in critical discussions by the providers. These positive excerpts indicated that these participants perceived quality care being delivered and the presence of an adequate level of support to meet their complex care needs.

“*She [physiatrist] doesn't miss a thing. She is very attentive. Will explain things in detail.*

*When she admitted me to Rehab for the two-week stay, my wife nicknamed her St.*

*Name…Because my wife was at her wit’s end trying to figure out what to do. And, uh the*

*doctor basically took that out of her hands and took over*.” (P006)

## Discussion

This study provides an overarching illustration of the patient journey of home care recipients from hospitalization to the return to the community and resumed home care service. The findings offer insights into the touchpoints, pain points, facilitators, and feelings experienced and perceived by these clients regarding their journey. We derived categories and themes from our data that highlighted issues with communication and continuity of care. All participants identified factors that mitigated negative transition experiences, such as relying on long-standing relationships and advocating for themselves. The findings contribute to the focus on the care transition needs of home care clients and the optimization of a more integrated health system.

In many countries worldwide, patients receiving home care services typically live with comorbid conditions, with high functional and cognitive impairment rates [30–32]. Sinn and colleagues observed that in Ontario, Canada, 7.3% of patients died, 16.6% were hospitalized, and 44.4% visited the emergency department within 90 days of being admitted to home care [30]. Unsurprisingly, most of the participants in our study had hospitalizations that resulted in significant functional decline and hospital-acquired infection. In this context, individuals leave the hospital with higher medical complexities, and they or their family members receiving home care require additional support to remain in their own homes. Our finding supports previous studies that describe the post-discharge period as a vulnerable time for these patients [33, 34]. More specifically, adaptation to life after the hospital is experienced as challenging when health problems compound daily activities such as laundry, meal preparation, and meeting basic needs like toileting [33].

During their transition journey, participants reported not feeling listened to and having limited information about their treatment and discharge plan while navigating between hospital-to-home. In a few cases, patients experienced harm that could have been prevented including loss of mobility, hospital-acquired infection, functional decline, and emotional damage. Further, adverse health outcomes contributed to delayed discharge from the hospital, resulting in additional usage of health care resources upon return home.

Hospital-based patient harm remains an ongoing issue. In Canada in 2021/22, 5.8% of patients experienced hospital harm during acute hospital admission [35]. Internationally, the pooled rate of hospital harm is 6% of patients across medical settings [36]. These findings are consistent with other research on communication failures between patients and health care providers [37] and the experience and impacts of hospital-based patient harm [38]. Similar to our findings, patient-provider miscommunication is often identified as a quality and safety concern by patients even without an adverse event [37]. Participants’ experiences of feeling unheard is detrimental, regardless if negative health outcomes or physical harm is caused.

Participants experienced challenges with the continuity of home care service providers, namely PSWs, once discharged home. The challenge stemmed from workforce constraints that resulted in either the home care organization being unable to resume service or the client needing assistance from multiple agencies to meet higher care demands. As a result of COVID-19, home care agencies faced extreme challenges in providing high-quality, in-home client care, including the adoption of virtual care use. The home care sector also experienced personal protective equipment shortages and staffing constraints caused by COVID-19 infection [39, 40]. These issues are compounded by unprecedented home and community care staffing shortages observed in a tripling of staff vacancies—a 331% increase in PSW openings from 2020 to 2021 [41]. Our findings show that home care clients not only value and desire consistency in home care service providers, but they also make sacrifices to achieve consistency in care. Despite critical issues of delivering continuity of care, there is limited literature exploring challenges of scheduling consistency for PSWs in home care delivery and pressures this causes for delivering high-quality care [10, 42]. Other research has detailed the importance of consistent PSWs in building trusting relationships with clients and families and delivering tailored care to match client needs and preferences [10, 43]. In our study, patients receiving homecare and their family caregivers described relational continuity with home care providers as an enabler of quality transitions. Participants also discussed continuity of care from home and community care workers prevented harm and contributed to a safety net in the community. The linkage between quality home care and continuity of care in the literature [44], and broadly speaking, relational continuity in health care has led to more effective and efficient diagnosis and management of health problems and a means of building patient trust [45]. In our study we found that when established home care services were disrupted by planned or unplanned hospitalizations and homecare was resumed, the disruption in relational continuity was destabilizing for clients and caregivers and may contribute to potential safety issues.

Both client and caregiver participants addressed needing to speak up for themselves when their needs went unmet. Across all participants, they leveraged their professional expertise or previous transition experiences to self-advocate. Advocating is a strategy for patients and families to engage in the care experience meaningfully. By doing so, they can make informed decisions, communicate effectively with providers, and build strong connections with others [46]. Our findings contributes evidence that confirms other research on advocacy [47]. Yet, uniquely these authors describe “growth” in families' ability to engage in “assertive advocacy” that allowed for collaborative partnerships rather than mistrust and conflict.

Our study has several important implications. Addressing improved patient-provider communication, the transitions warrant greater attention. Although this is not a novel finding, communication improvements could be considered through an integrated care lens. Opportunities exist in the home and community care sector to identify innovative, evidence-based practices that could enhance the continuity of care. One example is the renewed focus on health care co-production, which includes patients and families as influential members of the health care teams at all levels of service delivery—system, organizational, and one-on-one [48]. Home care offers ideal conditions for fostering participation in health care co-production, given that relational continuity, interprofessional collaboration, and client desire in the direct care and care planning [11]. Research findings also revealed that scarcity of resources may have led to clients facing trade-offs in quality of care owing to concerns around consistency in care. This is an important finding as it contributes to our understanding of client-provider partnerships to maintain treatment preference and client safety. Furthermore, our study contributes to the patient journey mapping body of literature in two ways. First, it highlights the complexity of health care navigation experiences from the viewpoint of clients and caregivers, including areas of improvement. There is a clear need to optimize coordination and collaboration within and between sectors. A scoping review identified that interaction and communication between health care providers were key contributors to optimizing home care [49]. Second, our patient journey mapping approach via interview and follow-up interview provided a mechanism for participants to review and comment on the collective journey map. This could be an opportunity for further co-designing with end-users on targeted action that aligns with the care recipients' needs.

## Limitations

Our study has several limitations for consideration. While providing rich narrative data, findings from our study must consider the limitation of generalizing results to other populations and settings. However, our research has the potential value to be transferable whereby readers can discern the extent to which the findings are similar and different to their own situations [50]. Additionally, the number of participants that self-identified as interested in participating in the study was limited, and therefore the number of participants resulted in a small sample size of seven participants. Although guidelines on adequate sample size for patient journey mapping do not exist, studies commonly cite larger sample sizes as ten or more participants [13]. In contrast, research using patient journey mapping with large samples of hospital staff reported limitations from not gathering information from the perspectives of those with lived experience [51]. Participants in this study were homogenous, and a lack of diversity among study participants is a limitation. A more diverse sample such as participants from marginalized communities that do not speak English may provide more diverse experiences and lead to more generalizable findings. For example, research has shown that racism and racialization intersect with other aspects of identity and social determinants of health, including sexism, ageism, and ableism, to name a few [52]. We also acknowledge the potential for self-selection bias among participants, including those who cannot effectively advocate for a quality care transition and may have been excluded. Despite a homogenous small sample size, our study is strengthened by gathering rich descriptions of the lived experiences of home care clients and their care partners. Another area for improvement is how we captured the care transition journey itself. Participants were asked about the hospital discharge during the 2021 period; however, many also drew on other health care transition experiences, which may have led to a blurring interpretation. This flexible approach supports how patients and caregivers view their care transition experiences – not as discrete events but as a compilation of ongoing, intersecting transitional experiences.

## Conclusion

Our study provided insight into the hospital-to-home care transition experiences and perspectives of home care clients and their caregivers by mapping their collective journey. The patient journey map enabled a collective illustration of the care transition depicted in their touchpoints, pain points, feelings, and findings that can help identify potential improvement areas. Patient journey mapping offers an opportunity to demonstrate how care is experienced and acknowledges home care clients and their caregivers as critical partners that need to be involved in care delivery across the continuum.

## Data Availability

The dataset analyzed for the current study is not publicly available due to confidentiality guaranteed to the participants. However, data are available from the authors upon reasonable request, conditioned by the REB and participants' permission. If someone wants to request the data from this study, please contact the corresponding author, Marianne Saragosa, Marianne.saragosa@mail.utoronto.ca.

## Abbreviations

PS: Personal support
PSW: Personal support worker
